# Measuring the efficacy of anti-malarial drugs *in vivo: *quantitative PCR measurement of parasite clearance

**DOI:** 10.1186/1475-2875-9-312

**Published:** 2010-11-05

**Authors:** Khalid B Beshir, Rachel L Hallett, Alice C Eziefula, Robin Bailey, Julie Watson, Stephen G Wright, Peter L Chiodini, Spencer D Polley, Colin J Sutherland

**Affiliations:** 1 Faculty of Infectious and Tropical Diseases, London School of Hygiene & Tropical Medicine (LSHTM), Keppel St, London, WC1E 7HT, UK; 2 Hospital for Tropical Diseases, Mortimer Market Centre, Capper St, London, WC1E 6JB UK; 3 HPA Malaria Reference Laboratory, LSHTM, Keppel St, London, WC1E 7HT, UK

## Abstract

**Background:**

Artemisinin-based combination therapy, currently considered the therapy of choice for uncomplicated *Plasmodium falciparum *malaria in endemic countries, may be under threat from newly emerging parasite resistance to the artemisinin family of drugs. Studies in Southeast Asia suggest some patients exhibit an extended parasite clearance time in the three days immediately following treatment with artesunate monotherapy. This phenotype is likely to become a more important trial endpoint in studies of anti-malarial drug efficacy, but currently requires frequent, closely spaced blood sampling in hospitalized study participants, followed by quantitation of parasite density by microscopy.

**Methods:**

A simple duplex quantitative PCR method was developed in which distinct fluorescent signals are generated from the human and parasite DNA components in each blood sample. The human amplification target in this assay is the *β tubulin *gene, and the parasite target is the unique methionine tRNA gene (*pgmet*), which exhibits perfect sequence identity in all six *Plasmodium *species that naturally infect humans. In a small series of malaria cases treated as hospital in-patients, the abundance of *pgmet *DNA was estimated relative to the human DNA target in daily peripheral blood samples, and parasite clearance times calculated.

**Results:**

The qPCR assay was reproducibly able to replicate parasite density estimates derived from microscopy, but provided additional data by quantification of parasite density 24 hours after the last positive blood film. Robust estimates of parasite clearance times were produced for a series of patients with clinical malaria.

**Conclusions:**

Large studies, particularly in Africa where children represent a major proportion of treated cases, will require a simpler blood sample collection regime, and a method capable of high throughput. The duplex qPCR method tested may fulfil these criteria, and should now be evaluated in such field studies.

## Background

Artemisinin-based combination therapy (ACT) is currently considered the best treatment for uncomplicated *Plasmodium falciparum *malaria [1]. The artemisinin component of these combinations produces a characteristic rapid reduction in parasite biomass immediately after treatment, but these compounds are metabolized in hours, and thus combination with a partner drug is required to provide complete parasite clearance with short treatment regimens, and to minimize the opportunity for evolution of parasites resistant to either component drug [2,3]. Recently, however, there have been reports of a decline of ACT efficacy, manifested as slower parasite clearance than expected in a significant subset of patients receiving artemisinin monotherapy, and ACT, in Western Cambodia [4-6].

In areas where ACT is deployed as the major anti-malarial treatment option, early warning of any decrease in the rate of parasite clearance in treated patients would enable adequate countermeasures to be taken to prevent onward transmission of these potentially resistant parasites, and to facilitate the search for genetic markers associated with this newly recognized parasite phenotype. In an individual, the persistence of parasites to day 3 following chemotherapy is a reliable predictor of subsequent treatment failure [7], and thus a marker for this would assist in the rational use of alternative treatment regimens to prevent prolonged parasite carriage, and enhanced transmission of novel parasite genotypes [8].

Parasite Clearance Time (PCT) is estimated by measuring change in peripheral parasitaemia in a sequence of samples taken after treatment, typically for 72 hours, and currently this requires microscopic examination of blood films. However, accurate microscopic estimation of parasite density requires considerable expertise; false negatives can easily be read especially at low-level parasitaemia; it is labour intensive and suffers from low throughput. Further, as the majority of ACT-treated patients are free of parasitaemia by microscopy within 48 hours of treatment, many closely-spaced venous blood samples in catheterized in-patients have been taken in previous studies to obtain a useful estimate of PCT by this method [5,6]. This approach is not suitable for large clinical trials, particularly in African settings, where the majority of participants are likely to be outpatients under the age of 10 years; such excessive sampling would be considered unethical in most such studies. A simple, rapid method for estimating PCT that did not require multiple venous blood samples, but could be achieved with relatively few finger-prick blood samples, spotted and dried on filter paper for storage, would thus be a useful new tool for large African studies of anti-malarial efficacy. However, absolute quantitation of parasite density from dried filter-paper blood spots is difficult, as the volume of blood in each spot is usually not known, and the efficiency of DNA extraction and target amplification differs between paper types and even among individual patients [9].

This paper reports the development and use of a new duplex quantitative real-time PCR (qPCR) assay to measure parasite clearance after anti-malarial treatment. To overcome problems with absolute parasite quantitation in the intended future application to DNA extracted from dried filter paper blood spots, parasite DNA abundance is normalized to a human white blood cell DNA target in the same sample, permitting calculation of relative parasite density in each sequential patient sample by the delta-delta C_T _(ΔΔC_T_) method [10]. The time required to clear 95% of the parasites (PCT_95_), and the ratio in parasite density between admission and 48 hours post-treatment, or parasite reduction ratio (PRR_48_), are then easily derived. These parameters were used to compare treatment response among patients treated with different anti-malarial regimens at the Hospital for Tropical Diseases (HTD), London.

## Methods

### Samples and patients

Parasitized peripheral blood was obtained from malaria patients admitted to HTD, as part of routine in-patient care [11]. Samples were collected on admission and approximately every 24 hours until cleared from the peripheral blood after treatment. Written informed consent was given by patients 1, 2 and 3 for additional biological studies of malaria parasites isolated from their blood samples to be carried out, according to a protocol approved by the University College London Hospital (UCLH) Research Ethics Committee (ref. 07/Q0505/60). Parasite isolates from patient 4 were investigated in detail at the specific request of the attending physician, as a suspected case of quinine treatment failure.

For each sample, DNA was purified from an aliquot of 200 μL of peripheral blood collected in EDTA, using the Qiagen QIAamp blood extraction kit and was eluted in a volume of approximately 100 μl [11]. In addition, for patients 1, 2 and 3 between 2.5 mL and 4.0 mL of pre-treatment (day 0) leuco-depleted blood was prepared and DNA extracted using the Qiagen maxi extraction kit. *P. falciparum *genome sequence data was generated from these samples using the Solexa Illumina platform at the Wellcome Trust Sanger Institute (WTSI), Hinxton, Cambridgeshire. These data, for patients 1, 2 and 3, are freely available via the MapSeq software platform at WTSI, designated OX007, OX005 and OX006, respectively [12].

### PCT using microscopy

Microscopic examinations of thick and thin smears, stained by the rapid Field's method for diagnosis and estimate of parasite density, and Giemsa-stained for discrimination of *Plasmodium *species, were performed according to standard methods by at least two experienced microscopists. This was performed in the Department of Clinical Parasitology at HTD, a fully accredited diagnostic laboratory that undergoes external quality assurance as a participant in the UK National External Quality Assessment Scheme for Parasitology. Parasite density is expressed as the percentage of erythrocytes which carry asexual stages of *P. falciparum*. The density of *Plasmodium ovale curtisi *parasites in patient 1 was not specifically measured by the microscopists, but was estimated to be less than 1% of total parasitaemia, *P. falciparum *being dominant at all time points.

### Designing primers and probes

Specific primer pairs and double-labelled fluorescent hydrolysis probes were designed using Primer Express software (AP Applied Biosystems) to amplify part of the *Plasmodium *tRNA methionine (PgMET) gene, which exhibits 100% sequence identity between *Plasmodium vivax *and *P. falciparum *(Genbank accession numbers: AL844509, nt 1575551-1575622; AZ570363, nt 127-56). Primers based on these sequences successfully amplified a fragment of *pgmet *from *P. ovale curtisi, Plasmodium ovale wallikeri *[13]*, Plasmodium malariae *and *Plasmodium knowlesi *DNA. The identity of the PCR target was confirmed by direct sequencing for each species. Thus it was concluded that this amplicon was suitable for cross-species parasite quantitation, under the assumption that each species had a single copy of the *pgmet *gene.

The human β tubulin gene (HumTuBB; Genbank accession no. NM_030773) was chosen as the endogenous normalizing gene for human white blood cell DNA, and a suitable PCR amplicon with internal probe sequence was identified using Primer Express. The primer and probe sequences for both PgMET and HumTuBB are given in Table 1.

**Table 1:** Amplification primers and double-labelled hydrolysis probes for PgMET and HumTuBB.

Primer/Probe	5' Fluorophore	Sequence	3' Quencher
PgMET_F1		5'-TGAAAGCAGCGTAGCTCAGA	
PgMET_R2		5'-CGCGTGGTTTCGATCCACG	
PgMET_pB	FAM	5'-GGGGCTCATAACCCCCAGGA	BHQ2
HumTuBB_F2		5'-AAGGAGGTCGATGAGCAGAT	
HumTuBB_R2		5'-GCTGTCTTGACATTGTTGGG	
HumTuBB_Joe	JOE	5'-TTAACGTGCAGAACAAGAACAGCAGCT	BHQ2

A 20 μl PCR master mix was prepared as follows: 5.5 mM MgCl_2 _buffer (Bioline), 0.3 mM dNTPs (Bioline), 0.3 μM forward and reverse primers, 0.2 μM of each probes (MWG-Biotech), 10× NH4 buffer (Bioline), 1.0 unit of BIOTAQ™ DNA polymerase (Bioline) and molecular grade water. 5 μl DNA was added and a total of 25 μl reaction was run in a Rotorgene 3000 thermocycler (Corbett, Australia). A cut off of 40 cycles was used to define positive samples. International Standard (INT) for *P. falciparum *DNA [14], which contains white blood cell DNA from the original donor specimen, was used as a positive control while parasite negative blood and water were used as negative controls. All reactions were performed in triplicate. The amplification and florescence detection was performed with the following two-step conditions: 95°C for 6 min; 40 cycles of 95°C for 15 s and 68°C for 1 min.

## Results

### Case descriptions

Patient 1 (OX007) presented with symptomatic malaria at HTD after returning from attending school in Lagos, Nigeria, and was found to harbour a mixed infection of *P. falciparum *(at 3.2% parasitaemia) and both gametocytes and trophozoites of *P. ovale curtisi *(at less than 0.03% parasitaemia), confirmed by PCR. The patient was treated with intravenous (i.v.) quinine (QN) for 1 day, oral QN for 5 days and given a full dose of sulphadoxine-pyrimethamine on discharge.

Patient 2 (OX005) developed symptomatic malaria after visiting Ghana, presenting to a local clinic in outer London one week after symptoms began. Unfortunately, treatment was further delayed due to a misdiagnosis of pandemic influenza (H1N1). The patient was seen again after four days and was immediately hospitalized, with the following signs of severe falciparum malaria: renal function impairment, hepatic function impairment, acidosis, antigen test for *P. falciparum *positive. The patient was started on i.v. QN, and blood films were taken and sent by courier to HTD. A diagnosis of falciparum malaria was confirmed by the on-call parasitology biomedical scientist, with a parasitaemia of 3%. The patient's condition had further deteriorated and transfer to the Intensive Care Unit (ICU) at HTD was arranged. Therapy with i.v. QN was continued for five days, and metabolic derangements gradually improved under ICU management. By the fourth day of treatment, only gametocytes of *P. falciparum *were observed on blood films. Five sequential daily blood samples were available.

Patient 3 (OX006) spent two weeks in Kenya based in Nairobi, and visiting Lake Naivasha, an area considered low risk for malaria. On advice, no prophylaxis was taken. The patient presented at night to a hospital outside London, and was antigen test positive for *P. falciparum*. Blood-films were taken and immediately couriered to the on-call parasitology biomedical scientist who identified extreme hyperparasitaemia of *P. falciparum *(approximately 2 × 10^6 ^parasites μl^-1^; 39% parasitaemia). To ensure early commencement of treatment, prior to transfer of the patient, the same motorcycle courier was dispatched in haste back to the referring hospital with i.v. artesunate (AS), which was administered just prior to departure for HTD. The patient was transferred to ICU, and treatment with i.v. AS continued. The patient exhibited the following signs of severe malaria: severe acidosis, renal impairment, deranged hepatic function, respiratory distress and haematuria. Remarkably, the patient remained conscious and lucid throughout his period of care in ICU. Haemofiltration was required for renal failure, but the initially high parasitaemia fell rapidly under treatment. AS was given up to day 5, followed by a full oral course of doxycycline. The patient was transferred from ICU to a standard medical ward on day 5, and discharged on day 7. Six sequential samples were available for analysis.

Patient 4, a British oil industry worker, presented with uncomplicated falciparum malaria in Port Harcourt, Nigeria, while working as a contractor in Nigeria and Angola. Quinine therapy administered in Port Harcourt failed to resolve a reported high parasitaemia, and the patient was transferred to HTD, London, by his employer, as a precaution. The patient reported previous bouts of malaria, but dates were not specified. *P. falciparum *infection was confirmed, with an initial parasite density of 0.5% on arrival in London. Oral QN therapy was continued at HTD for 48 hours (five doses in total) but poor response was again observed, necessitating a switch to oral atovaquone-proguanil (AP) (four tablets daily, for three days). Five sequential daily blood samples were available for analysis. The patient was clinically well by day 4 and was discharged before complete parasite clearance, with the remaining AP dose to be taken upon arrival in Nigeria.

All patients responded to treatment and were discharged after clinical improvement and reduction of parasite density to at or near zero.

### Microscopic estimates of parasite density

**Figure 1:**
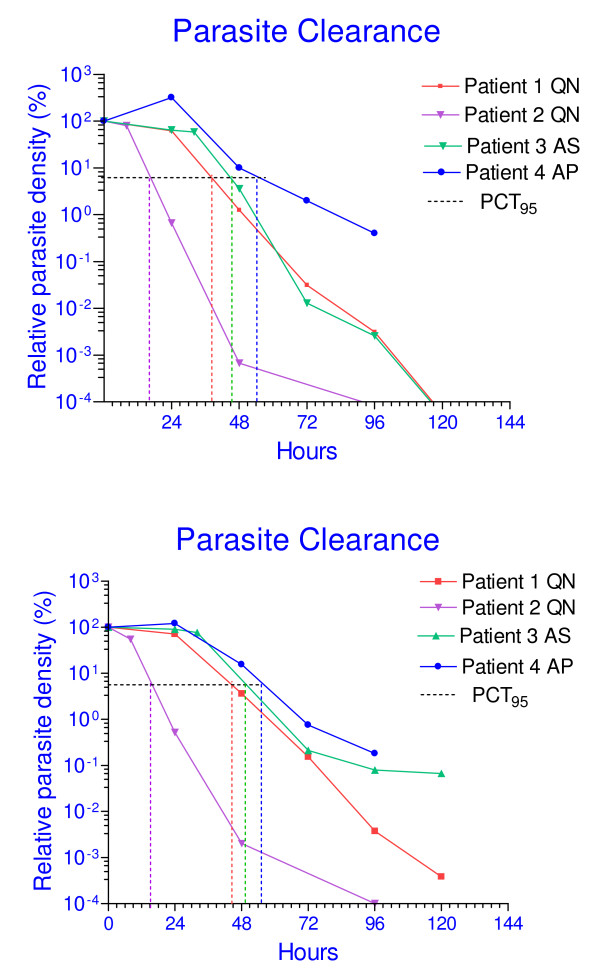
**Comparison of PCT Estimates from daily microscopy versus qPCR.** Upper panel depicts daily microscopy data. Initial parasite density was converted into % of pre-treatment level. Patient 3 had very high initial parasitemia (39%). The poor response of patient 4 over the initial 48 hr of QN treatment led to substitution with AP.  Lower panel depicts qPCR data, analysed using the ΔΔCT calculation. Relative density of parasite DNA at each sample time point was converted to percentage of  starting abundance in order to compare it with microscopy.

Estimated parasite density for each patient before and during treatment is shown in Table 2. PCT was extrapolated from microscopy data for each patient, and estimates of PCT of 120 h, 96 h and 120 h were obtained for patients 1, 2 and 3, respectively. There were insufficient data points to estimate PCT for patient 4 by microscopy. Due to the microscopic limit of detection (50 parasites/μl), PCT estimated from microscopy data does not give accurate results when the parasitaemia is very low and thus an additional parameter, the time to clearance of 95% of the starting parasites, PCT_95_, was also estimated. Treatment response is partially dependent on admission parasitaemia and this was taken into account by estimation of the parasite reduction ratio over 48 hours, PRR_48h_, for each patient. A log graph of relative parasite density versus time was plotted in order to compare PCT for each patient (Figure 1a). The PCT_95 _values for the four patients were 49, 16, 45 and 56 hours respectively.

**Table 2:** Parasite density by microscopy of daily blood samples from each patient

Parasite ID	Origin	Day 0	Day 1	Day 2	Day 3	Day 4	Day 5	Day 6	Treatment
1 (OX007)	Nigeria	3.2	2	0.004	0.001	0.0001	0	ND	Quinine
2 (OX005)	Ghana	3	2.4	0.02	0 (G)*	0 (G)	ND	ND	Quinine
3 (OX006)	Kenya	39*	25	24*	1.4	0.005	0.001	0	AS
4	Nigeria/ Angola	0.5	1.6	0.05	0.01	0.002	ND	ND	Quinine, AP

Densities are given as percentage parasitaemia (i.e. percentage of total erythrocytes counted that are infected) (G) denotes presence of gametocytes.

ND - not done, as patient previously discharged.

* samples were not available for qPCR

### Estimating PCT by qPCR

**Figure 2:**
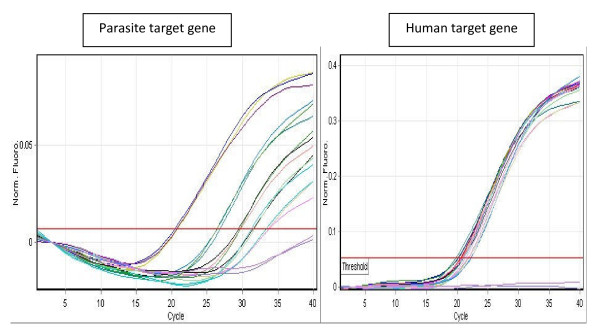
**Real-time amplification plot from duplex qPCR**. Real-time amplification plot of sequential samples (run in triplicate) from patient 2 at 0, 24, 48, 72 and 96 hours after QN treatment using primers for the parasite target gene (left) and the human normalizer gene (right). Each trio of curves represents DNA amplification from peripheral blood of patient 2 taken at a different time point.

Amplification of the HumTuBB gene was used to normalize the qPCR for the amount of infected blood analysed in the reaction. Amplification of both PgMET and HumTuBB was optimized for efficient hydrolysis probe detection of both targets in the same assay tube. Figure 2 shows raw data output of the qPCR assay, as fluorescent signal in each of the two channels, versus cycle number and a threshold set empirically by reference to positive and negative controls run in the same assay. The number of cycles of amplification of the human gene required to cross the threshold level of fluorescence (cycle threshold value, C_T_) remained stable throughout post-treatment follow-up, while the parasite gene showed a daily decrease as parasite density fell under treatment.

Cycle Threshold (C_T_) values were imported into a Microsoft Excel spreadsheet for analysis. Difference in C_T _(ΔC_T_) of the parasite target gene (PgMET) and the C_T _of the human reference gene (HumTuBB) of the same sample, was calculated as follows:

$$\Delta {\text{C}}_{\text{T}}=\Delta {\text{C}}_{\text{T}(\text{PgMET})}-\Delta {\text{C}}_{\text{T}(\text{HumTuBB})}$$

The change in ΔC_T _over time following treatment (ΔΔC_T _) was computed as:

$$\Delta \Delta {\text{C}}_{\text{T}(\text{n})}=\Delta {\text{C}}_{\text{T}(\text{n})}-\Delta {\text{C}}_{\text{T}(0)}$$

where n is time after treatment and time 0 is defined as the time at which the pre-treatment diagnostic sample was taken, or if that was unavailable, as for patients 3 and 4, the time at which the first sample in our series was taken.

The parasite density in each sample relative to the calibrator sample (day 0) was then estimated using the formula 2^-ΔΔCT ^[10]. The relative parasite abundance was plotted over time (Figure 1b). The qPCR plots are similar to those based on microscopy data (Figure 1a) but provide additional quantitative data in the later samples (days 3, 4 post-treatment) due to the higher sensitivity of qPCR.

**Figure 3:**
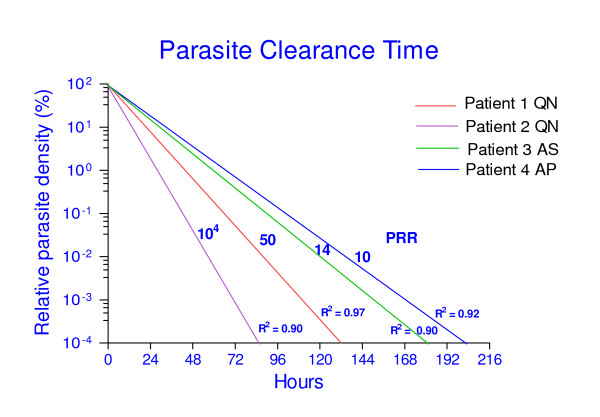
**Best fit log-linear parasite clearance curves for each patient**. The graph shows the Parasite Reduction Ratio (PRR) for each anti-malarial treatment, estimated from qPCR data. PRR is the ratio in parasite density between admission and 48 hours post-treatment. R^2 ^(coefficient of determination) reflects the goodness of fit of the log linear regression.

The natural log of relative parasite abundance against time was plotted for each patient and fitted well with a simple linear model. Using this model of the parasite clearance dynamics in each patient, we again calculated PCT (with clearance defined as parasite density at 10^-4^% that of the starting sample, i.e. a reduction of 10^6^-fold), PRR_48 h _and PCT_95 _parameters (Figure 3). These are compared to parameter values derived directly from microscopy and qPCR data in Table 3. For patients 1, 2 and 4 there is good agreement between the PRR_48 h _and PC_95 _parameter values estimated directly from microscopy or qPCR. Direct observation of PCT by qPCR was not possible for these patients, as all were parasite positive in this assay at the time of discharge, even though negative by microscopy. However, clearance time estimates derived from extrapolating the log-linear model to 10^-4^% of starting parasitaemia also agreed well with microscopic observations, and this was true whether microscopic or qPCR data were used to derive the line of best fit (Table 3). The one exception was patient 3, in that an observed clearance time by microscopy of 120 h in this individual, and a log-linear extrapolation from these data of 121 h, contrasts with a much longer clearance time of 183 h from qPCR data extrapolated to 10^-4^% of starting parasitaemia. This suggests that caution is needed in applying this methodology to estimate PCT, as opposed to reduction ratios, in patients with severe malaria presenting as hyperparasitaemia.

**Table 3:** Comparison of measured and estimated parameters of parasite clearance

A. PCT (hr):					
**Patient (ID)**	**Drug**	**Micr**. ***observed***	qPCR* *observed*	**Micr**. ***LL***	**qPCR** ***LL***

**1 (OX007)**	QN	120	-	122	134
**2 (OX005)**	QN	96	-	87	89
**3 (OX006)**	AS	120	-	121	183
**4**	QN; AP	-	-	220	199

**B. PRR_48 h _(ratio, no units):**					

**Patient (ID)**	**Drug**	**Micr**. ***observed***	qPCR * *observed*	**Micr**. ***LL***	**qPCR** ***LL***

**1 (OX007)**	QN	80	30	100	50
**2 (OX005)**	QN	100 000	100 000	25 000	25 000
**3 (OX006)**	AS	50	14	100	14
**4**	QN; AP	10	7	10	10

**C. PCT_95 _(hr):**					

**Patient (ID)** **Drug**	**Drug**	**Micr**. ***observed***	qPCR * *observed*	**Micr**. ***LL***	**qPCR** ***LL***

**1 (OX007)**	QN	49	44	33	38
**2 (OX005)**	QN	16	16	15	15
**3 (OX006)**	AS	45	49	39	56
**4**	QN; AP	56	55	60	54

PCT Parasite Clearance Time

PRR48h Parasite Reduction Rate - ratio between admission count and 48 hours later

PCT95 (hr) the time required to clear 95% of parasites

LL log linear model for best fit

* All samples reported negative by microscopy were positive by RT-PCR and hence PCT was not directly observed by RT-PCR

## Discussion

The current study presents the design and validation of a rapid, highly sensitive and potentially high-throughput assay to detect and quantify malaria parasites. Compared to expert double-read microscopy, the assay successfully detected and accurately measured malaria parasite densities in four individual patients treated with different anti-malarial regimens. The sensitivity, accuracy and reproducibility of the PCT assay have been further validated against the WHO International Standard for *P. falciparum *DNA, which is essentially lyophilized whole blood from a malaria patient, and thus provides both human and parasite targets for our duplex qPCR [14]. The assay can detect *Plasmodium *densities as low as five parasites per μl of peripheral blood; parasite DNA was detected in two of the post-treatment samples that were reported negative by microscopy, and in a third sample reported to harbour scanty gametocytes of *P. falciparum*. This appears to be the first real-time PCR assay specifically designed to measure parasite clearance in follow-up of treated malaria patients, and has distinct advantages over previous methods used for this purpose, such as sybrG detection of 18 S ribosomal DNA sequences [11]. The *pgmet *target has the advantage of being equally suitable for amplifying all *Plasmodium *species, and quantifies total parasite load in multi-species infections. It is a relatively simple matter to adapt the assay to any specific single parasite species of choice.

The assay was designed specifically for use with dried blood-spot samples collected simply in field situations, without measuring blood sample volumes, which would then be transferred to a laboratory with qPCR capacity. As a first step to validating this approach, the sensitivity, specificity and quantitative accuracy of the assay has been demonstrated here in a UK reference laboratory for parasitology, against high quality microscopic diagnosis. The efficacy of anti-malarial treatment is typically assessed by monitoring the clearance of parasites from the peripheral blood, and the speed at which symptoms and signs resolve. The utility of the assay to compare anti-malarial treatment response in different patients in this small pilot study has been demonstrated, and clear differences shown in PCT, PRR_48 h _and PCT_95 _among the four patients (Table 3, Figure 3). The ability to discern details of parasite clearance dynamics in individuals demonstrates the likely utility of this assay for detection of *P. falciparum *isolates with reduced sensitivity to ACT treatment. The qPCR method is now being successfully used to estimate PCT and PRR_48 h _using DNA extracted from dried filter paper blood spots. These have been collected during follow-up of a randomised efficacy study of two ACT regimens in 300 children with malaria in Mbita, western Kenya in 2009, similar to earlier studies in this area [15].

Dondorp and colleagues [6] used closely-spaced microscopic examination of blood films in small groups of malaria patients to demonstrate unusually slow parasite clearance time in some *P. falciparum *infected individuals from western Cambodia treated with AS. This parasite clearance phenotype could be explained by heritable changes in parasite growth rate, but may also indicate evolution of new mechanisms of resistance to artemisinin compounds [16,17]. The duplex qPCR assay, capable of relatively high throughput, and thus incorporation in larger studies, should assist in performing association studies to identify candidate genetic markers in *P. falciparum *of the artemisinin slow clearing parasite phenotype. Such markers, once validated, would be of enormous benefit in monitoring and containing resistance to ACT therapy.

Study protocols are now being developed that incorporate the qPCR PCT assay as an endpoint in large scale clinical trials of ACT efficacy in Africa. Initial testing of the assay on filter-paper DNA has shown promising results, and thus there is optimism that the method can be adopted to detect the phenotypic characteristics of parasites in clinical trials during the three days immediately after treatment. As the proportion of positive microscopy readings for ACT-treated patients on day 3 is generally less than 3% [7], a particular advantage of the qPCR PCT assay is its potential to detect and quantify parasites in many more patients on that day (Figure 3).

It is perhaps not surprising that the performance of the qPCR assay was markedly different in patient 3, who presented with extreme hyperparasitaemia, but recovered under AS monotherapy. There appeared to be a persistence of parasitized erythrocytes as detected by PCR for longer, and at greater estimated densities, than was expected from the microscopy results. It is unlikely the presence of peripheral gametocytes were responsible for this qPCR result, as the microscopists did not indicate the presence of sexual stages of *P. falciparum*, an observation routinely recorded. The higher parasite density estimated by qPCR could be due to the well-described circulation of more mature parasite forms, with significantly higher DNA content per cell, in severe malaria. Dead parasites may also contain detectable parasite DNA, but are not expected to circulate in a patient with an intact spleen, although pigment-positive phagocytes may also contain some detectable parasite DNA in parasites with very high parasitaemia. Dead parasites appear to be of no consequence in comparisons of qPCR-derived parasite densities with microscopy in uncomplicated *P. falciparum *infections in which parasite density is in the normal range [11]. Thus, it is expected that the qPCR assay will be most useful in studies of treatment response in uncomplicated malaria patients in the absence of hyperparasitaemia.

## Conclusions

This study provides proof of principle that a duplex fluorescent qPCR assay can be deployed to robustly estimate the rate of parasite clearance in treated malaria patients. The particular assay used here has the advantage of deploying a parasite target sequence that is identical in all *Plasmodium *species investigated and thus provides an estimate of total parasite burden. The method could be easily adapted to use specific amplification targets for each *Plasmodium *species.

## List of abbreviations

ACT: artemisinin combination therapy; AP: atovaquone-proguanil; HTD: Hospital for Tropical Diseases, London; HumTuBB: human b-tubulin gene sequence; ICU: intensive care unit; PCT: parasite clearance time; PgMET: *Plasmodium *genus methionine tRNA gene sequence; PRR: parasite reduction ratio; QN; quinine; qPCR: quantitative (real-time) PCR

## Competing interests

The authors declare that they have no competing interests.

## Authors' contributions

KB designed the PCT assay, carried out experiments and wrote the first draft of the manuscript. RLH designed the assay, processed samples, and contributed to writing the second draft of the manuscript. ACE, RB, SW and PLC were responsible for patient care, and contributed to the second draft of the manuscript. JW coordinated microscopy, and oversaw quality control and sample storage in the parasitology laboratory. SP conceived of the PgMET assay, performed initial validation experiments, processed samples and contributed to the final manuscript draft. CJS conceived of and coordinated the study, processed samples and wrote the final draft of the manuscript. All authors read and approved the final manuscript.
